# Development of a training programme for home health care workers to promote preventive activities focused on a healthy lifestyle: an intervention mapping approach

**DOI:** 10.1186/s12913-015-0936-7

**Published:** 2015-07-09

**Authors:** Maaike E. Walters, Arie Dijkstra, Andrea F. de Winter, Sijmen A. Reijneveld

**Affiliations:** 1 Division of Community & Occupational Medicine, Department of Health Sciences, University Medical Center Groningen, P.O. Box 196, 9700 AD Groningen, The Netherlands; 2 Department of Social Psychology, University of Groningen, Grote Kruisstraat 2/1, 9712 TS Groningen, The Netherlands

**Keywords:** Healthy eating, Home health care, Intervention mapping, Lifestyle, Nurse, Older adults, Physical activity, Prevention, Training programme

## Abstract

**Background:**

Lifestyle is an important aspect in maintaining good health in older adults, and home health care (HHC) workers can play an important role in promoting a healthy lifestyle. However, there is limited evidence in the literature regarding how to develop an effective training programme to improve the physical activity level and fruit and vegetable consumption of older adults within a HHC setting. The aim of this paper is to describe how Intervention Mapping (IM) was used to develop a training programme to promote preventive activities of HHC workers relating to the physical activity and fruit and vegetable intake of older adults living at home.

**Methods:**

IM, a systematic theory and evidence-based approach was used to develop, implement and evaluate the training programme. This entailed a literature search, a survey, semi-structured interviews and consultation with HHC workers and various field experts, and a pilot training session.

**Results:**

The determinants associated with the provision of preventive activities were identified, and an overview was created of those objectives, matching methods and practical applications that could influence these determinants. The performance objectives for the HHC workers were early detection and monitoring, promoting a healthy lifestyle, informing colleagues, continuing allocated preventive activities and referring to other experts and facilities. Findings were translated into a comprehensive training programme for HHC workers focused on motivating older adults to adopt and maintain a healthier lifestyle.

**Conclusions:**

IM was a useful tool in the development of a theory-based training programme to promote preventive activities by HHC workers relating to fruit and vegetable intake and physical activity of older adults.

## Background

Lifestyle is a key factor in good health; this is also true for older adults [24]. Sufficient physical activity and fruit and vegetable intake can have a beneficial effect on the maintenance of good physical and mental health [25, 38, 40, 42, 49]. Furthermore, physical activity and nutrition programmes can result in, respectively, increased physical activity and positive nutrition-related outcomes [3, 45]. The promotion of physical activity and fruit and vegetable intake is an important aspect of enhancing the health of older adults, however, research shows that the fruit and vegetable intake and physical activity pattern of many older European adults could be improved [13, 14, 22, 47].

Home health care (HHC) could prove an important setting for interventions here. HHC in the Netherlands is a form of nursing care provided at home, comparable to nursing practices at home in e.g. France and Germany. This care can be given, for example, to clients with chronic illnesses, clients with a handicap or to older adults to support ADL. Nurses conduct more simple care tasks, e.g., washing clients, or more complex medical nursing tasks, e.g., setting insulin dosages, depending on their level of nursing education. HHC workers are a valuable channel for providing preventive activities because they are in frequent contact with their clients and can therefore observe changes and developments in the clients’ behaviour, but evidence on the effectiveness of these activities lacks. Another advantage is that they observe their clients in an environment that is safe for the clients: their own homes. Furthermore, a relationship of trust often forms between HHC worker and client, which makes it easier for the HHC worker to raise the subject of lifestyle.

Interventions in the HHC context show promising results, for example positively affect health and functional status for a diversity of interventions provided at home [29–31, 43] and the topics studied include prevention of malnutrition, prevention of falls and prevention of pressure ulcers [8, 29, 34, 36]. Although prevention in the area of lifestyle entails quite different interventions and requires different behaviour from the HHC worker than other areas of prevention, these results support the hypothesis that HHC workers could also play an important role in providing prevention to promote physical activity and healthy eating.

The provision of preventive activities by HHC workers is low and could therefore be improved (unpublished manuscript submitted for publication). In order to achieve this, it is first necessary to describe the different preventive activities that HHC workers can execute to promote physical activity and healthy eating. These activities can be divided into six areas: early detection and monitoring, informing, promoting, continuation, referring and recording. Early detection and monitoring means detecting health risks such as inactivity or the risk of obesity. Informing means discussing the advantages of healthy and disadvantages of unhealthy behaviour with the client. Promoting entails motivating the client to be more physically active and to eat more healthily. Continuation means continuing to provide the activities that colleagues have been providing or to follow up with new activities. Referring entails informing clients about opportunities in the area if extra help is needed to change their behaviour. The last area, recording, means registering the health of clients in their health records. All these preventive activities are executed in the context of the individual client, meaning that the HHC worker always is sensitive to the individual needs of the client and inquires what the client can and cannot do by himself. For example, when it concerns dietary habits, the clients’ needs and wishes are mapped, as well as who is doing the shopping and who prepares the meals. As HHC workers have not previously perceived lifestyle prevention to be among their duties, they consequently do not provide it. Intervention is therefore necessary to encourage them to provide such preventive activities.

There is limited evidence in the literature regarding how to develop an effective training programme, specific within a HHC setting, to improve the physical activity level and fruit and vegetable consumption of older adults. Intervention Mapping (IM) was therefore used for the process of developing a training programme [5, 6]. As IM has not previously been used in the HHC context, the purpose of this article is to describe how it was used to develop a training programme to promote preventive activities by HHC workers relating to the physical activity and fruit and vegetable intake of older adults living at home. This is the first study describing the development of such a program within the HHC context focused on physical activity and fruit and vegetable consumption.

### Intervention mapping

IM was used to develop this training programme [5, 6]. It consists of six consecutive steps that help identify needs and find solutions by integrating theory and taking into account empirical findings from relevant literature and information collected from the target population, see Table 1 [7]. Since 1998, IM has been widely used by several researchers who found it to be a fitting, useful, systematic and practical framework [20, 26, 33].

**Table 1:** Intervention Mapping Protocol used for the training programme development

IM step	Aim	Methods
1. Needs assessment	Analyse the target group (older adults), their prevention needs and the accountable enabling determinants and barriers	Literature search
		Survey in a comparable area
		Consult project management group and interview local field experts
2. Programme objectives	Define the determinants and formulate the performance and change objectives that need to be addressed within the training programme	Survey
		Literature search
		Consult project management group and interview local field experts and HHC workers
3. Theory-based methods and practical applications	Select the theory-based methods and practical applications that will influence individuals and their environment	Literature search
		Consult project management group and interview HHC workers
4. Programme plan	Define the components and materials of the training programme and materials and conduct a pilot to pre-test it	Consult project management group and interview local field experts
		Pilot training for HHC workers
5. Programme implementation	Develop a plan to achieve solid programme adoption and implementation	Literature search
		Consult homecare organization management
		Collect ideas from HHC workers and training instructors for implementation
6. Evaluation plan	Define the method and measuring instruments to evaluate the effects of the training programme and the process development and check whether the programme needs adjustment	Evaluation by HHC workers (questionnaire and discussion) and training instructors (discussion)

*Source*: [8, 36]

Below we first describe the most important groups of that are involved: The HHC clients as the target group, and the HHC workers as the channel. In the subsequent steps of Intervention Mapping, information needs to be gathered systematically from different types of sources, using different methods. These methods (e.g. literature search; survey; interviews) are next described below. The Medical Ethical Committee of the University Medical Center Groningen evaluated the study protocol and considered it was not necessary to file it for ethical approval.

### Setting, target group and HHC workers

#### Setting and target group

The training was developed for HHC workers within an HHC organization in a deprived, rural area of the Netherlands. This deprived area entails many inhabitants with a low socioeconomic position (SEP), with more unhealthy behaviours, more chronic diseases and a lower life expectancy compared to other regions of the Netherlands. The target group, clients of the HHC workers, were community-dwelling, dependent older adults who receive home care. Clients with various health problems of differing severity were taken into account, with the exception of those with mental diseases such as dementia and those who receive palliative care.

#### HHC workers

The HHC workers with whom and for whom the training will be developed worked in the selected areas and included nursing aides and registered nurses who provide care at home for the clients the most. These HHC workers provide their clients with basic and medical and nursing care. Their common task also includes prevention, which can mean detecting emerging problems such as pressure ulcers, malnutrition, obesity, falling, problems with medication use, depression and incontinence [41].

## Methods

### Literature search

A first means of gathering information to develop the training programme is a literature search. This was conducted in EBSCOhost and ScienceDirect to find relevant information from other studies that could be integrated into the training. Various combinations of the keywords lifestyle, physical activity, exercise, fruit, vegetables, diet, preventive care, preventive activities, prevention, health promotion, psychosocial determinant, nurse, homecare, elderly, older adults, intervention, programme and training were used. The search began in 2011, and updates were carried out until June of that year. Only articles written in English were taken into account.

Furthermore, while following IM, suitable theoretical methods and practical applications were chosen that could influence the relevant determinants. A theory-based method is a technique or process used to influence the determinants of behaviour. Applications are techniques that use theory-based methods in a way that suit the intervention population and context [5, 6]. In an early stage of the development of the intervention, a training was chosen as the channel to change the behaviour of the HHC workers. The reason for this was the complex nature of the specific behaviour to be influenced and the need for the HHC workers to practise the preventive activities.

### Survey

Second, a survey was conducted in one of the work areas of the HHC workers to generate an overview of the enabling determinants and barriers that might be associated with the provision of preventive activities. The analyses of this survey showed that social support, social pressure, role modelling and knowledge were associated with the performance of preventive activities. These potentially modifiable factors have been taken into account when developing the training programme. Forty-six HHC workers, not included in this study, response 40 %, used a self-report questionnaire to rate whether they conducted twenty-eight types of preventive activities relating to physical activity and healthy eating. They were also asked about what motivated them to provide these preventive activities. More information can be found in the study of Walters, Dijkstra, de Winter and Reijneveld (unpublished manuscript submitted for publication).

### Interviews and consultation with HHC workers and field experts

Third and prior to the development of the training, semi-structured interviews and meetings were held with local field experts to gather information about which tasks undertaken by the HHC workers related to prevention together with the possible determinants that could influence them. These experts were district managers of the HHC organization, a medical officer, a senior staff member of the community, a sports consultant, a manager of a social work foundation and a general practitioner. In addition, five semi-structured interviews were conducted with HHC workers to discover what motivated them to engage in preventive activities and what could be possible barriers for them not to perform these activities. This information was used to further tailor the training programme to the HHC workers. Verbal consent was obtained for all interviews.

### Pilot training

A final way for input for the training development was a pilot study. A pilot training was set up to pre-test several aspects of it. This pilot training was evaluated and minor modifications were made to the training protocol.

### Theoretical model

#### Integrated model of change (I-change model)

The I-change model [17] explains motivational and behavioural change, and it was applied to identify possible determinants of conducting preventive activities by HHC workers. The I-Change Model describes that hidden and apparent behaviours are determined by a person’s intention or motivation to perform a specific type of behaviour. It is an integrated model that combines several theories [18], such as the ASE model originating from the Theory of Reasoned Action [23, 37], the Transtheoretical Model [37], the Social Cognitive Theory [4] and the Precaution Adoption Model [48]. The key concepts are that it ensures that potential influenceable determinants related to the target problem are taken into account and that the best applicable theoretical methods and applications are used to address these determinants. Several possible determinants, according to the model, were taken into account in developing the training, such as awareness, knowledge, self-efficacy, attitude, skills and social support.

## Results

In this paragraph, the results of the six steps of Intervention Mapping are presented.

### Step 1: Needs assessment

The purpose of this step was to analyse the problem faced by the target group, older adults. The problem of an unhealthy lifestyle accounts for our target group, older adults living in a rural part of the Netherlands, the region of Groningen. Approximately 60 % of older adults (65+) in the province of Groningen are overweight and 37 % do not meet the Dutch physical activity recommendation (30 min of physical activity at least 5 days a week). In addition, approximately 67 % do not meet the recommended intake of vegetables (200 g a day) and 50 % do not meet the recommended two pieces of fruit a day [10]. These numbers are comparable for older adults in other parts of the Netherlands. A low socioeconomic position (SEP) seems to explain much of the unhealthy lifestyle of older adults in this area. A high rate of 80 % of adults between 50 and 64 years of age with a low SEP are overweight, but for older adults with a high SEP this is much lower, namely 50 %. Of those adults with a low SEP who are 65 years old and over, 62 % are overweight, whereas the rate for those in this age group with a high SEP is 46 % [10]. Thus, this aspect should be taken into account when developing the training programme.

Action is therefore needed to change the unhealthy behaviour patterns of these older adults in region of Groningen and encourage them to adopt a healthier lifestyle. The study focused on the HHC workers providing preventive activities to promote a healthy lifestyle. It seems that, despite their potential influence, the HHC workers do not make full use of this opportunity. The literature tells us that only half of all nurse practitioners provide preventive counselling on physical activity on most days of the week, and only a third of physicians discuss nutrition or diet with their patients [12, 15]. Furthermore, Walters, De Winter, Reijneveld and Dijkstra (unpublished manuscript submitted for publication) found that HHC workers only engage in a limited number of preventive activities relating to physical activity and healthy eating.

There could be several explanations for the behaviour of the HHC workers. Psychosocial factors, such as level of knowledge, could have an effect on their behaviour, and work-related factors, such as the work culture within the organization and facilities such as a reporting tool, could also be associated with the provision of preventive activities. These factors will be further elucidated in step 2.

### Step 2: Formulating programme objectives

In this step, specific determinants that could change the target behaviour were defined, and the programme goal and corresponding objectives formulated. This overall goal was divided into five separate performance objectives and translated into change objectives based on the needs assessment, literature search, survey and interviews with HHC workers and experts in the field (see Table 2). In Table 2, change objectives are stated how to change the behaviour of the HHC workers to stimulate HHC clients to engage in more physical activity and healthier eating behaviour. These performance objectives describe what the HHC workers need to do to promote the target behaviour, namely provide preventive activities. The change objectives in Table 2 state what needs to be done to accomplish the performance objectives.

**Table 2:** Overview of performance and change objectives for the various determinants

Performance objectives	Determinants					
	Awareness	Knowledge	Self-efficacy	Attitude	Skills	Social influence
Early detection and monitoring	Is aware of which activities he /she does and does not provide with regard to early detection and monitoring of health risks	Knows how to recognize risks in the client	Feels confident that he/she can detect health risks in the client at an early stage	Believes that early detection of health risks in the client is an essential start to prevention to improve the lifestyle of the client	Possesses the skills to detect risks in the client at an early stage	Supports and is supported by colleagues and supervisor in the early detection of certain health risks in the client
Promoting a healthy lifestyle	Is aware of which activities he /she does and does not provide with regard to promoting a healthy lifestyle	Knows what constitutes a healthy lifestyle and how to communicate this to the client	Feels confident about his/her own skills and knowledge in promoting a healthy lifestyle	Is positive about the benefits for the client of promoting a healthy lifestyle	Possesses the communication skills necessary to promote a healthy lifestyle	Supports and is supported by colleagues and supervisor in addressing and promoting a healthy lifestyle in the client
Informing colleagues	Is aware of which activities he /she does and does not provide with regard to informing colleagues about allocated prevention	Knows how to use the report tool to ensure the optimal continuation of prevention	Feels confident in using the report tool to inform colleagues about allocated prevention	Believes that informing colleagues about allocated prevention is beneficial	Possesses the skills to use the report tool to inform colleagues	Supports and is supported by colleagues and supervisor in informing colleagues about allocated prevention
Continuing preventive interventions	Is aware which activities he/she does and does not provide with regard to continuing the allocated preventive activities	Knows how to continue to provide the allocated preventive activities	Feels confident that he/she can ensure the continuation of preventive activities	Believes that continuing preventive activities is essential in effecting behaviour change in the client	Possesses the skills to ensure the continuation of preventive activities	Supports and is supported by colleagues and supervisor in continuing to provide preventive activities to the client
Referring to other experts/facilities	Is aware of which activities he/she does and does not provide with regard to referring to other experts and facilities	Has knowledge of referral possibilities in the area and the procedure of referring to other experts	Feels confident about his/her knowledge of how and when to refer to other experts and facilities	Believes that referral to other experts/facilities can be vital for the client in some situations	Possesses the skills to refer the client to other experts and facilities	Supports and is supported by colleagues and supervisor in referring clients to other experts and facilities

The five performance objectives were:

- The HHC workers detect and monitor any risks or potential risks, such as obesity, inactivity and decreased autonomy. These activities can be performed on a regular basis.
- When the HHC workers are at the clients’ home, they communicate the risks to the client and promote a healthier lifestyle. For example, HHC workers ask how older adults in their opinion can increase their physical activities or promote active participation in household activities.
- The HHC workers inform their colleagues about the preventive activities they have provided by recording their preventive activities during each contact.
- If behaviour is to change, continuity of care is needed dependent on the degree of behaviour change the client already achieved. The HHC workers therefore continues to provide preventive activities adjusted to the individual client.
- If the HHC workers need help with prevention, they should request this from a colleague or supervisor. If they require specific help, they may need to refer clients to another expert. If necessary, the HHC workers can refer clients to facilities with a focus on physical activity or healthy eating.

In order to meet these five objectives, some insight into the determinants of the behavioural objectives and psychological causes would be needed and is provided by the following literature search. The literature revealed that the health behaviour of the HHC workers themselves affects whether they engage in certain preventive activities: for example, McDowell, McKenna and Naylor [32] found that HHC workers who were physically active regularly were more likely to promote physical activity themselves. Another possible determinant was that HHC workers have not considered the provision of preventive activities relating to physical activity and healthy eating to be one of their duties. Other studies have revealed various barriers to providing preventive activities, namely lack of client interest and lack of time during a client visit [11, 16, 27, 44], lack of guidelines and educational materials [21, 28], and lack of specific training for health care workers on how to advise clients [21, 27]. The studies also provided examples of facilitating factors for the specific preventive activity of counselling: client motivation, family support and relationship with the client [27]. The attitude and self-efficacy of the HHC workers also seemed to be associated with comparable activities such as counselling [1, 9], and it was possible that their skill level was also of influence, which meant that the determinant skills also needed to be taken into account.

The various field experts (see methods) believed that the following were important determinants for the provision of preventive activities: the HHC worker’s level of knowledge, the quality of the relationship between the HHC worker and the client, the HHC worker having only a limited focus on health and a limited focus on health within the area. An important determinant that arose in the interviews with the HHC workers and was also found in the literature was a lack of client interest in changing their behaviour [11, 27]. The interviewees also believed that the following preconditioning factors would facilitate the provision of preventive activities: integrating time for prevention into their working hours and standardizing working methods to promote communication between colleagues.

In addition, the survey showed that the determinants social support, social pressure and modelling were associated with whether HHC workers provide preventive activities relating to physical activity and healthy eating. This accounted for the total provision of preventive activities but also for specific activities such as informing the client, promoting a healthy lifestyle and continuation and recording of activities.

Once the above performance objectives and relevant determinants had been defined the following psychosocial determinants were selected that could be influenced or considered a preconditioning factor. Additional determinants from the I-change model that might influence whether the HHC worker provides prevention activities, such as attitude, self-efficacy and awareness, were also included. This yielded the following determinants that could be influenced (see Table 2):

- awareness
- knowledge
- self-efficacy
- attitude
- skills
- social influence.

### Step 3: Theory-based methods and practical applications

In order to realize the performance objectives specified in step 2, suitable theoretical methods and practical applications were chosen that could influence the relevant determinants. Various methods were derived from the literature and selected to reinforce the provision of preventive activities [1, 2, 4], namely:

- self-reflection: reflecting on one’s own thoughts
- persuasive communication: using new argumentation and removing false notions
- positive reinforcement: giving positive reinforcement to reward desired behaviour
- feedback: receiving and giving personal feedback on behaviour
- information delivery and processing
- support from work organization: social support from colleagues and supervisors
- instruction: issuing clear instructions about the desired behaviour
- guided practice: practising good communication skills
- modelling: demonstrating desired behaviour so the HHC worker can model this
- goal-setting: setting clear goals for behaviour change
- tailoring: tailoring all of the above methods to the individual HHC worker.

These theory-based methods were the basis for a training programme for HHC workers. The aims and content of the training programme are listed in Table 3 together with the details of the practical applications. The preconditioning factors for the HHC workers to succeed in providing preventive activities were assured by creating a supportive culture throughout the entire HHC organization and embedding regular team meetings. This implementation plan is described in step 5.

**Table 3:** Determinants, methods and practical applications of the training^a^

Determinants	Theory-based methods	Practical applications
Awareness	Self-reflection	Introductory inspirational talk/group discussion/written assignment/feedback on home assignment
	Persuasive communication	Overall aim of the training programme is stated: ensuring client autonomy by enhancing lifestyle and focusing on physical activity and fruit and vegetable intake
		Exploration of which preventive activities workers already engage in with regard to physical activity and fruit and vegetable intake
	Positive reinforcement	Feedback on home assignment and experiences of starting a dialogue with the client about lifestyle
Knowledge	Information delivery (passive learning)	Lecture/written assignment/written information/game of statements
	Information processing (active learning)	Early detection of risk behaviour in the client
		Guidelines for physical activity and fruit and vegetable intake in older adults
		How to manage client barriers
		Presentation of theory of communication skills, Stages Of Change and written information in the training
		Knowledge of several disorders and recommended nutrition and physical activity for these
		Information on facilities and possibilities for clients to enhance lifestyle so that the HHC worker can advise them
Self-efficacy	Feedback/Instruction	Written assignment/group discussion/lecture/practical assignment
	Modelling	Exploration of the preventive activities the HHC workers have provided and their experiences during training sessions and work meetings Reflection on how the HHC workers deal with complex issues and how colleagues cope with this HHC workers will develop a plan of actions they will undertake in the client setting
	Goal-setting	
Attitude	Information delivery (passive learning)	Lecture/game of statements
	Information processing (active learning)	The importance of prevention focused on physical activity and intake of fruit and vegetables is explained
	Feedback	Feedback on the efforts the HHC workers have made and compliments on successes during training sessions and work meetings
Skills	Modelling	Learn from colleagues by observing them practising communication skills
	Guided practice	Practising communication skills with colleagues using the information given in the lecture
Social influence	Support from work organization	Group discussion/practical assignment/policy/protocol
		Exchange experiences in providing prevention during work meetings
	Modelling	HHC worker and colleagues explore which preventive activities they already provide in the area of physical activity and fruit and vegetable intake
		Integrate the process of engaging in preventive actions in the HHC organization

^a^Workers were fully involved in the development of the content of the training programme and the teaching strategies used

### Step 4: Programme plan

The practical applications were combined to form a training programme consisting of two components. The main component was a training to help HHC workers improve the preventive activities they provide during client visits. The second component was to focus on the topic of preventive activities relating to healthy eating and physical activity during work meetings.

#### Training

The training was developed in close cooperation with the HHC workers, the project management group and training instructors. Various parts of the training were pre-tested in a pilot training session. The training consisted of three sessions and was carried out over a timespan of 6 months. This number of sessions and the time span were chosen for two reasons. Firstly, the training was tailored to the HHC workers level of prior knowledge: All HHC workers have professional education and the present training was developed to be grounded in their prior knowledge and skills. Secondly, in the context of the HHC work organisation this was feasible concerning the available resources.

We took into account the various barriers which were discovered in earlier studies, namely lack of client interest and lack of time during a client visit, lack of guidelines and educational materials, and lack of specific training for health care workers on how to advise clients. To address the clients lack of interest, motivational interviewing was added to the training programme, making the HHC worker capable of informing the client of the importance of the health benefits that can be obtained by behavioural change and motivating the client to adjust his behaviour. The training programme aimed at acquiring skills to perform preventive activities within the time the HHC worker has with a client. The preventive activities can partly be performed during other usual HHC tasks. The lack of guidelines, educational materials and specific training was addressed by developing this training programme and corresponding tools.

The aim of the first training session was to help the HHC workers gain insight into the preventive activities they already provided and where there was room for improvement. They were also provided with early detection and monitoring tools, a health-related behaviour checklist and a registration tool, in order to enhance their knowledge of this topic. These tools were developed in the earlier survey study done by Walters et al. (unpublished manuscript submitted for publication). The health-related behaviour-checklist and registration tool were developed to support the HHC worker in providing preventive activities. The checklist concerns a list of various preventive activities the HHC worker can perform, whereas the registration tool is used as a monitoring tool for the HHC worker to register the agreements reached with the HHC client. In addition, various situations in which client behaviour seemed difficult to change were discussed. It was important that the HHC workers learned how to determine the clients’ personal goals in order to motivate them to change their behaviour.

The second training session was about practising motivating the client to adopt a healthier lifestyle. During this training session their experiences, both successes and failures, were discussed and were taken into account to further develop their knowledge and skills. The HHC workers were trained in how to converse with clients about their lifestyle and how to do this in a supportive way using motivational interviewing and persuasive communication. They also learned how to determine which stage of change the client was in and therefore adapt the motivational preventive activities to this specific stage.

The third training session focused on motivating the client to continue with a healthier lifestyle, even after a relapse. The HHC workers also learned how to give and receive feedback to and from colleagues and supervisors so that preventive activities can be discussed in work meetings. The HHC workers also discussed how to continue providing preventive activities and how to deal with barriers to this. See Table 3 for further details on the various training sessions.

The materials developed for the three training sessions consisted of a lifestyle checklist, registration tool and training booklet. The HHC workers can use the lifestyle checklist as a communication tool for addressing lifestyle with the client, and the registration tool to ensure continuity and as an aid in monitoring the preventive activities they have allocated. These tools are added to the client’s health file. The training booklet contains practical assignments that the HHC workers carry out during the training as well as the theory on communication with clients and how to overcome barriers clients may encounter when attempting to change their lifestyle.

#### Work meetings

In addition to the training programme, the HHC workers and their supervisors attended work meetings every 6 weeks. The topic of prevention was on the agenda at these meetings, and the HHC workers could discuss the preventive activities they provided and address any barriers they faced. Their colleagues and supervisor could offer any advice or share any experiences. These meetings also meant that the HHC workers received feedback and thus felt supported in the provision of preventive activities, which encouraged them to keep up the good work.

### Step 5: Programme adoption and implementation

The adoption and implementation of this training programme was kept in mind to ensure that it could be applied to the field of study and in future to a different field or community adoption of the program is crucial for example for the creation of support and ownership of the workers for the training. According to Rogers [39], the rate of adoption of an innovation (the intervention) depends on several characteristics: relative advantage, compatibility, complexity, trialability and observability. If the innovation has a higher relative advantage, compatibility, trialability and observability, and is low in complexity, it will be adopted more quickly than other innovations [39].

During the training programme, several important aspects became apparent that need to be taken into consideration. For example, it was important to involve the HHC workers in the programme adoption and implementation phase. This was achieved by discussing any ideas and recommendations that the HHC workers might have during the training sessions and taking them into account when developing the adoption and implementation plan. For example, the HHC workers indicated the way how they wanted to register the provided preventive activities and thus gave input for the registration tool. This ensured that the training programme was optimally tailored to the HHC workers and based on their own input. Moreover, involving the HHC workers in this process seemed to motivate them more to make a contribution and start providing prevention activities themselves. To ensure the full involvement and commitment of the HHC organization and allegiance of the intervention implementation, meetings were held with the management staff of the organization about the provision of prevention and the training. To further reinforce the implementation, supervisors discuss the provision of prevention with the HHC workers.

An inventory was also made of barriers, facilitators and ideas for the implementation suggested by the HHC workers, training instructors and management team of the HHC organization. It is very important that the supervisor of the HHC workers pays regular attention to the programme and the topic of lifestyle. This together with the assurance of continuity can be a standard item on the agenda of work meetings.

### Step 6: Evaluation plan

For the evaluation of the training programme, a longitudinal study with a quasi-experimental design will be carried out that will measure any changes in the behaviour of the HHC workers and their clients. The training development process will also be evaluated. This will show us how effective the training programme has been, and whether changes need to be made or the programme discontinued should the effects prove counterproductive. In this longitudinal study we aim to include two hundred HHC workers and three hundred HHC clients. A comparison will be made between two groups, a trained HHC worker group, and their HHC clients, and an untrained HHC worker group, and their HHC clients. Intermediate and final outcomes will be measured both at the level of the HHC workers and of their clients. The HHC workers will evaluate the programme at the end of each training session, both in a discussion and on an evaluation form. In addition, the training instructors, supervisors of the HHC workers, the management team of the home health care organization and the project management group will evaluate the training at evaluation meetings, in which they consider the value of each part of the training. This information can be used to adjust the training during and after the implementation of the programme.

The HHC workers and their clients will also evaluate the effect of the training programme at three time points in time: before the programme begins (baseline) and 6 and 12 months after the baseline measurement. The HHC workers and clients will fill in a questionnaire that asks them about the preventive activities they have provided or received. In addition, the level of physical activity and dietary pattern of the clients and satisfaction of the received preventive activities will be measured by questionnaire at these three points in order to determine changes in behaviour and satisfaction with the received training. The information obtained will be used to evaluate the training programme and decide whether changes need to be made and whether the programme as a whole should be continued. A control group will be added to the study to create a quasi-experimental design.

## Discussion

The aim of this article was to describe the use of IM to develop a training programme to promote preventive activities by HHC workers relating to the physical activity and fruit and vegetable intake of older adults living at home. We presented the methods used and the results of Intervention Mapping steps and how information was gathered to build the program. IM was chosen because it has been widely used by several researchers, although not used in a HHC setting and community-living older adults, who found it to be a fitting, useful, systematic and practical framework [20, 26, 33]. The training programme consisted of three sessions and aimed to enhance the provision of preventive activities by HHC workers by influencing the determinants awareness, knowledge, self-efficacy, attitude, skills and social influence. In short, the training programme was about making the HHC workers more aware of which preventive activities they already provided and where there was room for improvement, practising how to motivate and guide the clients in adopting a healthier lifestyle and encouraging them to continue with this healthier lifestyle. In addition to this training, the HHC workers could discuss which preventive activities they provided and address the barriers they faced at work meetings, because the topic of prevention had become a fixed item on the agenda.

IM proved to be a helpful tool in developing a training programme tailored to the needs of the target population, in this case HHC workers. The training programme was based on theory, matching methods and practical applications. IM facilitated the identification of those determinants that needed to be influenced to ensure behavioural change. The use of IM during the process of intervention development also created the opportunity to adjust the training programme during the implementation phase. Using IM in the development of a training programme meant that it was systematically developed, theory-driven and based on the available evidence. Interventions developed on the basis of the theory are often more effective when they target determinants of behaviour and behaviour change [35]. In the case of this study this meant conducting a survey and a thorough literature search beforehand, which provided an understanding of the facilitating determinants, barriers and preconditioning factors that influence the preventive activities. In addition, the training programme was culturally appropriate and tailored to the needs of the participants because the HHC workers were involved in its development. The training programme and its effect will also be evaluated by the HHC workers and their clients, which means that information will be obtained from various viewpoints. A further strength of IM is that it has already been successfully applied in many different settings within the field of health promotion as well as to various behaviours and populations [5, 6].

Other studies describing the development of a training programme also reported that IM was a helpful, beneficial or valuable tool [19, 20, 46, 50]. Zwerver, Schellart, Anema, Rammelo and van der Beek [50] developed a training course to teach insurance physicians how to apply guidelines for the treatment of depression, and IM proved to be a useful tool here. Van Rijssen, Schellart, Anema, De Boer and van der Beek [46] developed a communication skills training course for physicians, and found the IM protocol to be beneficial, despite the amount of time needed to develop an intervention. Another study found IM to be a valuable tool because it resulted in a training programme that matched the needs of the HHC workers and could be adapted to any changes [19]. Detaille, van der Gulden, Engels, Heerkens and van Dijk [20] used IM to tailor an existing training programme to employees with a chronic somatic disease. Although they did not take the perspective of the employer into account and did not evaluate whether the training programme met their needs, they still found IM to be a helpful tool, this time in tailoring an existing training programme, because using IM to develop a new intervention was too time-consuming [20].

Although IM is a valuable tool, it nevertheless has certain constraints that should be mentioned here. As IM is so broad and thorough, it makes training development a long and time-consuming process. While defining the multiple IM steps, constraints such as lack of information became apparent, for example information about the intensity and frequency of a training programme and how to implement and adopt it. In addition, the programme addressed a selection of risk behaviours, physical activity and dietary pattern, instead of multiple risk behaviours such as smoking or alcohol misuse. It is not yet clear which strategy works best. IM was selected for the development of the training programme, but there are other frameworks for developing behavioural interventions. It is therefore recommended that more extensive studies be conducted that take into account the aforementioned points.

This study has several strengths. Multiple methods were used, such as a survey of HHC workers, interviews with field experts and a pilot training session in the development of the training programme. This was to make sure that sufficient information was available at the start of the programme development. As the stakeholders were involved, the training programme could be tailored to the HHC workers and thus ensure relatively smooth implementation in the organization.

In addition to the strengths, it is important to note some limitations to the training development. One of the tools used was a survey to specify which determinants needed to be influenced. This survey had a relatively low response rate (40 %), which could have biased the outcome of certain determinants associated with the provision of preventive activities. In order to limit the risk of bias, a wide range of methods were used to identify which determinants needed to be influenced, and the I-change model, which also takes into account the particular determinants, was applied. A further limitation was the lack of a pilot for the second training session and therefore the lack of feedback on this session. Nevertheless, each session was evaluated so that the upcoming sessions could be improved.

Future studies should focus on specifying which determinants should be influenced by the intervention. This is one of the most important steps of IM because it serves as a main hook for the intervention. It is therefore essential to use multiple sources to reveal which determinants need to be addressed in order to influence the behaviour. The training programme should be sufficiently generalizable for HHC workers within another HHC context, given that this involves similar duties and a similar rural area. Organizations are consequently encouraged to adjust this training programme to their specific setting before implementing it. However, the next step is to implement and evaluate this training programme. The benefits of providing the training and meetings for HHC workers are inherent to the expectation that it will contribute to lowering the problems among HHC clients. Further research is necessary to determine whether this training programme can also be applied to other roles in the care sector, to other contexts, at home or abroad. Sufficient financial resources are needed to implement a comprehensive programme, and those resources are not always available. Further studies are also needed to enhance the provision of preventive activities by HHC workers so that they are actively involved in promoting physical activity and healthy eating. Finally, HHC organizations should further review how to maintain the knowledge and skills of HHC workers, for example in a refresher course as well as the implemental aspects within the organization.

## Conclusions

Although extensive and time-consuming, we found IM to be a useful tool in the development of a theory-based training programme to stimulate HHC workers to provide preventive activities relating to the fruit and vegetable intake and physical activity of older adults with a low social economic position. Our next step is to evaluate the effectiveness of the training in a longitudinal study with a quasi-experimental design.

## Abbreviations

HHC: Home health care
IM: Intervention Mapping
SEP: Socioeconomic position
I-change model: Integrated model of change
